# Distance-depending transcriptome changes of pancreatic stellate cells in paracrine pancreatic ductal adenocarcinoma co-culture models

**DOI:** 10.1038/s41598-024-68148-6

**Published:** 2024-08-04

**Authors:** Anais Zourelidis, Bogusz Trojanowicz, Yoshiaki Sunami, Gerd Hause, David Vieweg, Jörg Kleeff

**Affiliations:** 1 Department of Visceral, Vascular and Endocrine Surgery, University Hospital Halle, Martin-Luther-University Halle-Wittenberg, Halle, Germany; 2 Biocenter, Martin-Luther-University Halle-Wittenberg, Halle, Germany

**Keywords:** Cancer, Gastrointestinal cancer, Pancreatic cancer

## Abstract

Pancreatic stellate cells (PSC) are one source of cancer-associated fibroblasts (CAF) and play, therefore, an essential role in pancreatic ductal adenocarcinoma (PDA). Paracrine signalling between PDA cells and CAF has been widely studied, yet external influences on paracrine crosstalk are poorly understood. This study aimed to gain a deeper insight into the communication of PSC and cancer cells under different co-culture conditions via analysis of PSC gene expression profiles. Two contactless co-culture models with tumor cells from the p48-Cre; lox-stop-lox-Kras^G12D/+^; lox-stop-lox-Trp53^R172H/+^ mouse model (KPC) and murine PSC separated through a microporous membrane and grown in different compartments (standard co-culture) or on different sides of the same membrane (inverse co-culture), were established. RNA-Sequencing analysis of PSC mRNA was performed 24 h and 72 h after co-culture with KPC cells. For selected genes, results were confirmed by quantitative RT-PCR and immunocytochemistry. Standard co-culture displayed 19 differentially expressed genes (DEG) at 24 h and 52 DEG at 72 h. In inverse co-culture, 800 DEG at 24 h and 2213 DEG at 72 h were enriched. PSC showed great heterogeneity in their gene expression profiles; however, mutually regulated genes of both co-cultures, such as VCAN and CHST11, could be identified. VCAN-protein–protein interaction-network analysis revealed several shared genes between co-culture models, such as SDC4 and FN1. In conclusion, PSC show a varying susceptibility to cancer cell signals depending on the co-culture method, with intensified transcriptome changes with closer proximity.

## Introduction

Pancreatic cancer is on the rise and thus has been estimated to become the third most frequent cause of cancer-related deaths in the European Union by 2025^1^. Late diagnosis of pancreatic ductal adenocarcinoma (PDA) often results in an advanced disease stage with limited therapeutic options^2^. Therefore, early detection and a better understanding of the mechanisms that lead to initiation and progression of PDA are needed. A dense hypo-vascular and desmoplastic stroma is characteristic of PDA. With limited diffusion and convection, it functions as a mechanical and molecular barrier against potential therapies^3^. Pancreatic stellate cells (PSC) represent only a small part of pancreatic cells in the non-diseased pancreas, yet they play an essential role in tissue homeostasis and maintaining tissue structure^4^.

Most importantly, PSC are believed to be one of the sources of so-called cancer-associated fibroblasts (CAF), which are recruited early during tumorigenesis. CAF can progressively impact tumor growth and modulate invasiveness, metastasis, and response to therapy in murine models^5^. However, complete depletion of CAF in mice resulted in a more aggressive tumor type and reduced overall survival. Therefore, tumor suppressive features of CAF are also discussed^6^. Although PDA is a molecularly heterogeneous disease, key mutations of the protooncogenic gene Kras occur in over 90%, and mutations of the tumor suppressor gene Trp53 can be found in over 50–70% of cancers^2^. The so-called KPC mouse model, first established by Hingorani et al.^7^, harbors Kras^G12D/+^ and Trp53^R172H/+^ mutations in the progenitor cells of the mouse pancreas. KPC mice develop invasive tumours after 2–3 months and mimic clinical features of the human disease like metastasis and cachexia. PDA co-culture models have been widely used to study the interactions between cancer cells and PSC^8–10^, yet these models often do not allow a separate analysis of cell types and do not focus on the distance of the cell types. Furthermore, a variety of complex processes are occurring at the cancer basement membrane, with PSC/CAF and cancer cells directly interacting. Even basement membrane destruction through PSC has been reported in in-vitro PDAC organoid models^11^.

Paracrine signalling is defined as secretion from soluble signalling molecules from one cell affecting another cell in the immediate surroundings via diffusion. However, the exact effective reach is not defined due to complex and varying circumstances in vivo. Different concentrations of signalling molecules and the specific features of secreted factors have been discussed as reasons for a variable operating distance^12^. This study aimed to investigate early paracrine communication between PSC and KPC cells and the early changes of the stromal components under different conditions, represented by two different co-culture models. Therefore, we established two spatially separated co-culture models, where the exchange of soluble molecules was enabled, but PSC-KPC contact was prevented. This setup allowed reliable extraction of PSC RNA with high purity and investigation of PSC in direct, contactless proximity to cancer cells, which offered a new approach to investigating PSC/CAF-cancer cell interactions.

## Methods

### PSC isolation

Mouse experiments were approved by the Martin-Luther University Halle-Wittenberg (Nr. K2aM3) and conducted according to the guidelines of the European Parliament 2010/63/EU and ARRIVE guidelines. PSC isolation was performed by Nycodenz gradient centrifugation as described by Apte et al.^13^ with slight modifications. PSC were isolated from pancreata of 7–10-week-old wild-type C57BL/6 J mice from Charles River Laboratories (Sulzfeld, Germany). Mice were euthanized by cervical dislocation, and the PSC isolation directly followed. Subsequently, pancreata were carefully dissected in sterile fashion using blade, forceps, and scissors. All connective and excess tissue was removed from pancreas, specimens were transferred in a tube with 30 ml HBSS (Gibco, UK, catalogue number (cn) 14175095) and transported to working cell bank on ice. For each isolation, 4–10 pancreata were pooled, considering an equal gender ratio to balance out any possible effects of sex. After dissection of the pancreas, an enzyme solution with 10% collagenase P (Roche, Germany, cn 11213857001), 10% pronase (Roche, Germany, cn 11459643001), and 1% DNase (Machery-Nagel, Germany, 740963) in 10 ml GBSS was injected at multiple sites of the tissue with a 26 Guage syringe. GBSS was prepared according to the published protocol by Apte et al.^14^. Pancreata were then digested within the remaining enzyme solution for 7 min in a shaking water bath at 37° C. Lids of tubes were sealed with foil to prevent contamination. After dicing up the tissues finely with a scalpel, another digestion for 20 min at 37° C followed. The mixture was filtered through a 100 µm cell strainer. The filter was washed twice with HBSS. The filtrate was centrifuged at 450 g for 6 min at 4° C. The supernatant was discarded, and the pellet was washed once with GBSS solution. The pellet was then resuspended with 4.75 ml GBSS with 0.3% BSA (Sigma, Germany, A9418), and 4 ml of a 28.7% solution of Nycodenz (Alere Technologies AS, Norway, cn 1002424) in GBSS without NaCl was added. Carefully, 3 ml of GBSS with 0.3% BSA was layered on the suspension. The sample was centrifuged at 1400 g for 20 min at 4° C without deceleration. The white fluffy layer in between the levels was collected using a pipette. Harvested cells were then washed in GBSS. The pellet was finally resuspended with DMEM low glucose medium (Sigma-Aldrich, Germany, cn D5546) supplemented with 40% Nutrient mixture F12 (Gibco, UK, cn N6658), 16% FBS (Sigma-Aldrich, Germany, cn S0615), 1% Amphotericin B (Sigma-Aldrich, Germany, cn A2942) and 1% penicillin/streptomycin (Gibco, UK, cn 15140122). The culture medium was changed between 24 and 48 h for the first time, depending on attachment of cells controlled via light microscopy.

### Cell culture

PSC were cultured in DMEM low glucose medium (Sigma-Aldrich, Germany, cn D5546) supplemented with 40% Nutrient mixture F12 (Gibco, UK, cn N6658), 16% FBS (Sigma-Aldrich, Germany, cn S0615), 1% Amphotericin B (Sigma-Aldrich, Germany, cn A2942) and 1% penicillin/streptomycin (Gibco, UK, cn 15140122). A few days after isolation, a medium without Amphotericin B was used. PSC were used for experiments between the first and second passages. The KPC cell line was provided by Bo Kong, MD, PhD (University of Heidelberg, Germany) and was derived from a female, 18 week old p48-Cre; lox-stop-lox-Kras^G12D/+^; lox-stop-lox-Trp53^R172H/+^ mouse model. KPC cells were cultured in DMEM high glucose medium (Sigma-Aldrich, Germany, cn D5796) supplemented with 10% FBS and 1% penicillin/streptomycin. KPC cells were used for experiments between the first and 15th passages. All cells were cultured at 37° C in 5% CO_2_.

### Co-culture

**Figure 1:**
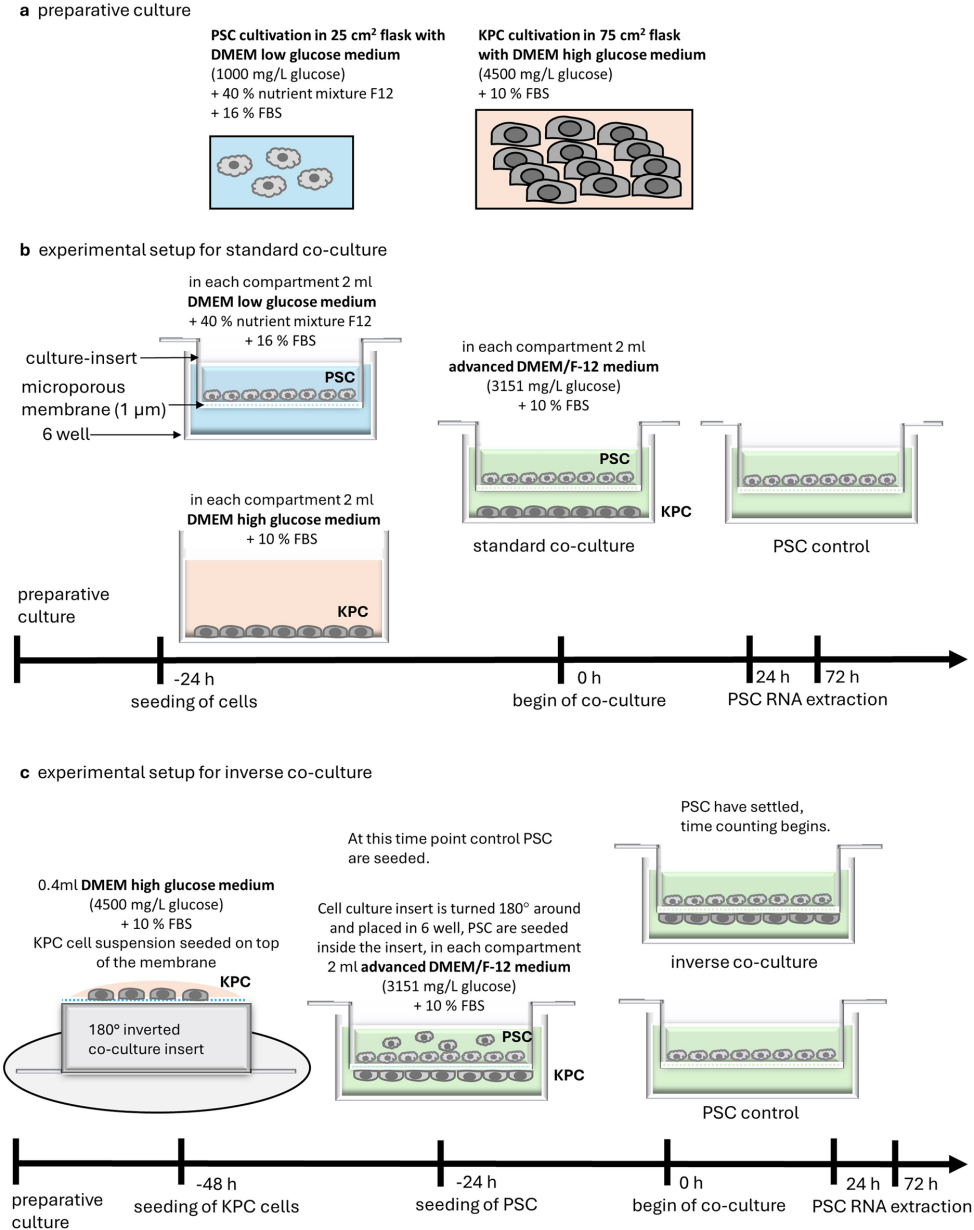
Schematic illustration of contactless co-cultures. Preparative culture of KPC cells and PSC (**a**). Schematic illustration of experimental setup for standard co-culture (**b**) and inverse co-culture (**c**). Each medium contained 1% penicillin/streptomycin.

For paracrine co-cultures sterile cell culture-inserts with a microporous membrane (Greiner, Germany) were employed. For preliminary co-cultures for electron microscopy imaging culture-inserts with 0.4 μm, 1 μm and 3 μm pores were used. Following experiments, including all co-cultures with extracted RNA, were conducted with a culture insert with a microporous membrane with 1 μm pore size. Sterile cell culture inserts with 0.4 μm pores (Greiner, Germany, cn 657640; Pore density: 1 × 10^8^ (cm^2^), membrane thickness: 22 ± 3 μm), 1 μm pores (Greiner, Germany, cn 657610; Pore density: 2 × 10^6^ (cm^2^), membrane thickness: 22 ± 3 μm) or 3 μm pores (Greiner, Germany, cn 657630; Pore density: 0,6 × 10^6^ (cm^2^), membrane thickness: 20 ± 3 μm) were used. The pore membrane material is polyethylenterephthalat (PET), the casing of the culture-insert is Polystyrene. Optical microscope images of the membrane surface show even distribution of pores (Fig. S1). In the standard co-culture PSC were grown in an insert with a porous membrane, separated from KPC cells growing on the bottom of the well allowing exchange of secreted messenger molecules via the culture medium (Fig. 1a,b). For standard co-culture, 24 h prior to the time counting 1 × 10^5^ KPC cells (up to passage 15) were seeded in the bottom of a 6-well dish, covered with 2 ml DMEM high glucose medium (supplemented with 10% FBS and 1% penicillin/streptomycin) and 1 × 10^5^ PSC from wild-type C57BL/6 J mice pancreata (passage 1–2) were seeded separately on top of the porous membrane of a culture-insert and covered with 2 ml DMEM low glucose medium (supplemented with 40% nutrient mixture F12, 16% FBS and 1% penicillin/streptomycin) while 2 ml of the same medium were filled into the lower compartment. Cells were counted with a counting chamber. After 24 h, the culture medium was changed to advanced DMEM/F-12 medium (Gibco, UK, cn 12634010), supplemented with 10% FBS and 1% penicillin/streptomycin, and the co-culture insert with PSC was transferred into the well with KPC cells. An amount of 2 ml medium each was used for each compartment. During the experiment, the culture medium was not changed anymore. For controls, PSC were seeded as well 24 h prior to time counting in the same manner as PSC for co-culture, the media change to advanced DMEM/F-12 medium, supplemented with 10% FBS and 1% penicillin/streptomycin was conducted at time point zero as well. A so-called inverse co-culture model was established to minimize the distance between the two cell types (Fig. 1a,c). In this model, KPC cells grew on the bottom, and PSC grew on top of the same porous membrane. For inverse co-culture, culture-inserts were turned around 180° and placed in a petri dish. Carefully, 0.4 ml of a suspension with 1 × 10^5^ KPC cells in DMEM high glucose medium, supplemented with 10% FBS and 1% penicillin/streptomycin was seeded on top of the membrane. A lid was placed over the construction and the petri dish was incubated for 24 h at 37 °C in 5% CO_2_. Thereafter, the attachment of the cancer cells to the bottom of the membrane was controlled via microscopy and the culture-insert was turned around 180° again. The insert was placed into a 6-well-dish and 1 × 10^5^ PSC were seeded into the culture-insert on top of the membrane. Both compartments contained 2 ml of advanced DMEM/F-12 medium, supplemented with 10% FBS and 1% penicillin/streptomycin. Another 24 h later, the time counting was started. For controls, PSC were seeded as well 24 h prior to time counting in the same manner as PSC for co-culture containing 2 ml advanced DMEM/F-12 medium, supplemented with 10% FBS and 1% penicillin/streptomycin in each compartment. During the experiment, the culture medium was not changed anymore.

To allow the same period for settling and attachment of PSC, the total time of PSC and KPC co-existence varied in between standard co-culture and inverse co-culture. Due to technical reasons, in inverse co-culture, KPC had to be grown prior to PSC seeding on the bottom of the membrane, therefore a direct co-existence started immediately after PSC were seeded. This led to a maximum co-existence time of 96 h in inverse co-culture instead of 72 h co-culture in standard co-culture.

### Electron microscopy

PSC and KPC cells were seeded as described above. After 72 h of co-culture, cells were fixed and processed for imaging. Therefore, cells were fixed with 3% glutaraldehyde (Sigma-Aldrich, Germany, cn 340855) in 0.1 M sodium cacodylate buffer (Roth, Germany, cn 51692) for 2 h and washed 2 × with sodium cacodylate buffer. Lipids were stained by a secondary fixation with 1% osmium tetroxide (Roth, Germany, cn 83711) in sodium cacodylate buffer for 30 min. Samples were washed 3 × with water and then dehydrated with 10%, 30%, and 50% ethanol (Fisher chemical, USA, cn 15802544) for each 30 min, followed by incubation with 70% ethanol and 1% uranyl acetate (Chemapol, Czech Republic) for 1 h and incubation with 70% ethanol overnight. After complete dehydration (90%, 100%, 100% for each 30 min) the samples were infiltrated with epoxy resin (ScienceServices, Germany, cn E14115) (Spurr, 1969^15^). Samples were incubated in ethanol: epoxy resin mixture for 3 h with a 3:1 ratio, 4 h with a 1:1 ratio, and overnight with a 1:3 ratio. This step was followed by incubation with 2 × pure epoxy resin for 8 h and polymerization for 12 h at 70° C. Samples were cut in 70 nm slices using an ultramicrotome (Ultracut R, Leica, Wetzlar, Germany), transferred to copper grids, and contrasted with uranyl acetate and lead citrate (Leica, Germany, cn 14450–60-3) with an EMSTAIN-appartus (Leica). The sections were analysed with an EM 900 transmission electron microscope (Carl Zeiss Microscopy, Germany). The acceleration voltage of the transmission electron microscope was 80 kV. Pictures were taken with a Variospeed D camera (SM-1 k-120, TRS, Germany) and ImageSP acquisition software (Unitary Enterpise "SYSPROG", Belarus).

### Immunostaining PDA

Human PDA tissue samples were obtained from patients undergoing surgery for PDA treatment. The study was approved by the ethical committee of the Martin-Luther University, Faculty of Medicine (Nr. 2019–037). All patients and persons involved in this study gave written informed consent. The study was performed according to the rules of the Declaration of Helsinki. Paraffin sections with human PDA were dewaxed with incubation in Neoclear (Sigma-Aldrich, Germany, cn 64741–65-7) for 20 min, followed by incubation in 100%, 96%, and 70% ethanol for 4 min, respectively, and incubation in distilled water for 4 min. To perform antigen retrieval, slides were heated in a microwave with a Tris (Roth, Germany cn AE15.1)-EDTA (Sigma-Aldrich, Germany, cn E1644-250G) solution (76.6%/ 23.4%; pH value 9) for 3 min at 700 watts, then for 7 min at 350 watts. Afterwards they were cooled down at room temperature, washed for 5 min with PBS (PanReac AppliChem, Germany, cn A0964,9010) and incubated with 1% BSA (Sigma, Germany, cn A9418) in PBS for 1 h. The anti-versican antibody (EPR23135-58, Abcam, UK) was diluted 1:500 with the Antibody Diluent (Agilent, USA, cn S0809). Slides were incubated with the antibody overnight at 4° C in a damp chamber. The following day, the slides were washed with PBS. The Dako EnVision + System-HRP Labelled Polymer kit (Agilent, USA, cn K4065) and the Dako liquid DAB + substrate chromogen system kit (Agilent, USA, cn K3468) were used for the following steps according to the manufacturer's instructions. The slides were counterstained with hematoxylin solution (Merck, Germany, cn 105175) diluted 1:5 in distilled water for 4 min. A final dehydration step was performed with incubation for 2 min in 96% ethanol and for 4 min, respectively, in 100% ethanol and xylene (Roth, Germany, cn 9713.1). Slides were covered with Entellan (Sigma-Aldrich, Germany, cn 107961) and cover slides. Slides were photographed with the Axioplan 2 microscope (Zeiss, Germany) and evaluated with AxioVision Software (Zeiss, Germany, SE64 Rel. 4.8).

### Immunostaining PSC

PSC were seeded in DMEM low glucose medium (Sigma-Aldrich, Germany, cn D5546) supplemented with 40% Nutrient mixture F12 (Gibco, UK, N6658), 16% FBS (Sigma-Aldrich, Germany, S0615) and 1% penicillin/streptomycin (Gibco, UK, cn 15140122) on a glass slide and incubated for 24 h at 37° C in 5% CO_2_. The slides with PSC were fixed with acetone (Sigma-Aldrich, Germany, cn 179124) at − 20° C for 10 min and then air-dried. Slides were incubated for 20 min in a 3% hydrogen peroxide (Merck, Germany, cn 107209)/methanol (Sigma-Aldrich, USA, cn 67–56-1) (1:5) solution at 4° C. Slides were washed with PBS and then incubated with the antibody overnight at 4° C in a damp chamber. All antibodies were diluted with the Antibody Diluent (Agilent, USA, cn S0809): 1:100 anti-alpha-smooth muscle actin antibody (17H19L35, Rabbit-monoclonal antibody, Thermo Scientific, Germany); 1:50 anti-vimentin antibody (clone 2I13, Rabbit-monoclonal antibody, Sigma-Aldrich, Germany); 1:500 anti-cytokeratin 19 (SA30-06, Rabbit-monoclonal antibody, Thermo Scientific, Germany); 1:1000 anti-GFAP antibody (ab7260, Rabbit-polyclonal antibody, Abcam, UK). As a negative control, PSC were incubated with PBS only. The following day, the slides were washed with PBS. The Dako LSAB2 system-HRP kit (Agilent, USA, cn K0609) and the Dako liquid DAB + substrate chromogen system kit (Agilent, USA, cn K3468) were used for the following steps according to the manufacturer's instructions. The slides were counterstained with hematoxylin solution (Merck, Germany, cn 105175) diluted 1:5 in distilled water for 3 min. Slides were covered with aquatex medium (Merck, Germany, cn 108562) and cover slides. Slides were photographed with the Axioplan 2 microscope (Zeiss, Germany) and evaluated with AxioVision Software (Zeiss, Germany, SE64 Rel. 4.8).

### Oil red O staining

PSC were seeded and fixed with 4% PFA in PBS (Merck, Germany, cn 100496). Slides were washed 1 × in PBS and then covered in freshly filtered and 5:2 in distilled water diluted oil red staining solution. For the oil red staining solution, 300 mg oil red O (Sigma-Aldrich, USA, cn O0625) was dissolved in 100 ml isopropanolol (Sigma-Aldrich, USA, cn 67–63-0). After washing the slides multiple times with distilled water, they were counterstained with hematoxylin solution (Merck, Germany, cn 105175) for 45 s. After repeatedly washing the slides in distilled water, they were covered with aquatex medium (Merck, Germany, cn 108562) and cover slides. Slides were photographed with the Axioplan 2 microscope (Zeiss, Germany) and evaluated with AxioVision Software (Zeiss, Germany, SE64 Rel. 4.8).

### RNA extraction

For RNA extraction at the respective time point, PSC in cell culture inserts were washed with 2 ml HBSS (Gibco, UK, cn 14175095), then 2 ml Trypsin–EDTA (0.05%) (Gibco, UK, cn 25300062) was added and let sit for 10 min at 37° C, controlling intermittently the detachment of cells microscopically. After 2 ml Advanced DMEM/F-12 medium (Gibco, UK, cn 12634010) medium was added, the suspension was transferred to a tube, and the insert was rinsed once more with 1 ml Advanced DMEM/F-12 medium, which was also added to the tube. The suspension was centrifuged at 336 g for 10 min at 23 °C. Afterwards, the supernatant was discarded, 300 µl Qiazol Lysis Reagent (Qiagen, Hilden, cn 79306) was added to the pellet, and the suspension was vortexed. For RNA extraction, a direct-zol RNA Microprep Kit (Zymo Research, Germany, cn R2061) was used, following the manufacturer's instructions. Finally, RNA was evaluated using RNAse-free water. The measurement of the RNA, which served for quantity determination as well as quality testing (A260/280 ratio ≈ 2), was carried out spectrophotometrically using NanoDrop One (Thermo Scientific, Germany).

### RNA-sequencing and data analysis

RNA sequencing was performed with n = 3 biological replicates per group. Library construction, sequencing, and bioinformatic analysis were performed by Novogene (UK). For each sample, a minimum amount of 100 ng RNA was submitted. Sample integrity and purity were verified with an agarose gel electrophoresis with the Agilent 2100 Bioanalyzer. For library construction, first mRNA was extracted from total RNA with poly-T oligo-attached magnetic beads. Then, mRNA was fragmented, cDNA was synthesized, and adaptors were ligated. Library preparation was performed using Novogene NGS RNA Library Prep Set (PT042). A library quality control with Qubit 2.0 Fluorometer Bioanalyzer, followed by quantification library quantification with qPCR and size distribution detection with Agilent 2100 was conducted. Illumina NovaSeq 6000 was used for sequencing and creating paired-end reads (150 bp). At least 20 million reads were read out per sample. CASAVA was used for base calling. Raw reads were processed through fastq, and therefore, reads containing adapters, reads containing > 10% bases that could not be determined, and low-quality reads (Qscore of over 50% bases of the read <  = 5) were removed. CG content distribution and base error rate were determined for a quality check. Subsequent analyses were carried out with the clean data. For mapping the reads to the reference genome, mus musculus (Genom ID ensembl\_mus\_musculus\_grcm38\_p6\_gca\ _000001635\_8), the HISAT2 algorithm (v2.0.5, parameter: –dta, –phred33) was used. Reads were quantified using FeatureCounts (v1.5.0-p3, parameter: default), and fragments per kilobase per million mapped reads (FPKM) of the genes were calculated. The threshold for an expressed gene was set at FPKM > 1. For differential gene expression analysis, the DESeq2 R package (v1.20.0, padj < 0.05) was used. Enrichment analysis, such as Gene Ontology (GO)- and Kyoto Encyclopedia of Genes and Genomes (KEGG)-analysis, was performed using the ClusterProfiler (v3.8.1, padj < 0.05) R package. PCA was conducted with the web tool GraphBio^16^. Heat maps were generated using Novosmart (Novogene, UK), an application developed on R shiny. Protein–protein-interaction networks were constructed by Novogene using and STRING protein interaction database^21^. Through blastx alignment (v2.5.0, evalue = 1e-10, max_target_seqs = 1), the interaction relationship of genes in STRING database was determined. PPI-Network files were then imported into Cytoscape software (v3.8.2)^17^ to visualize and modulated the network. For a reduced network, the confidence score cutoff in Cytoscape was set to 0.8 to obtain only high-quality results (80% confidence). RNA sequencing data is available at NCBI Gene Expression Omnibus (accession no. GSE264009).

### Quantitative RT-PCR

Selected genes were amplified through PCR (Rotor-Gene Q, Qiagen) using the primers listed in Table 1 and 5 × HOT FIREPol® EvaGreen® qPCR Supermix (Solis BioDyne, Estland, cn 08–36-0000S). For qPCR of one sample a mix of 4 µl 5 × HOT FIREPol® EvaGreen® qPCR Mix Plus, 1,5 µl antisense primer (10 µM), 1,5 µl sense primer (10 µM) and 11 µl RNase free water was prepared. All primers (100 µM) were diluted 1:10 with RNase free water. The total volume of one sample was 20 µl, therefore 2 µl cDNA was added to the above described 18 µl mix. Each sample underwent duplicate determination. After placing the tubes into Rotor-Gene Q cycler, the following steps were performed: hold 15 min at 95 °C, 40 cycles of 15 s at 95 °C, 20 s at 60 °C and 20 s at 72 °C. Data analysis was performed via Comparative Quantification^18^ (Rotor-Gene Q software, Qiagen, Germany, 2.3.5.). Primers were designed using the Primer-BLAST Tool (NCBI, Bethesda)^19^.

**Table 1:** Sequence of qPCR primers.

Primer		Amplificate size/ Primer length	Sequence
B2M mouse	as	150 bp/ 20 b	CAGTCTCAGTGGGGGTGAAT
	s	150 bp/ 20 b	ACGTAACACAGTTCCACCCG
RPL37A mouse	as	92 bp/ 21 b	AGAGGTGGTGTTGTAGGTCCA
	s	92 bp/ 21 b	CCAAGATGAAGAGACGAGCCG
VCAN mouse	as	169 bp/ 23 b	GGACCAAGTTCCACCCTGACAT
	s	169 bp/ 22 b	CTTCACTGCAAGGTTCCTCTTCT
CHST11 mouse	as	125 bp/ 22 b	CCTTCGGTGTGGACATCTGCTG
	s	125 bp/ 22 bp	TGTCACCTGGTCCCGTCTCATC
TIMP1 mouse	as	131 bp/ 23 b	TCTTGGTTCCCTGGCGTACTCT
	s	131 bp/ 22 b	GTGAGTGTCACTCTCCAGTTTGC
FN1 mouse	as	145 bp/ 22 b	CCCTATCTCTGATACCGTTGTCC
	s	145 bp/ 23 b	TGCCGCAACTACTGTGATTCGG
SDC4 mouse	as	92 bp/ 22 b	AGTCTGGCGGCTCGGATGACT
	s	92 bp/ 21 b	GGGCTCAATCACTTCAGGGAAG

### Statistical analysis

Each experiment was conducted with three biological replicates, for RNASeq replicates (n = 3) were sequenced individually, and then the mean was taken. The padjust value was adapted with Benjamini and Hochberg’s method^20^ for controlling the false discovery rate. A gene with a Padjust value < 0.5 was determined to be statistically significant expressed. A GO-term or KEGG-pathway with a Padjust value < 0.5 was determined to be statistically significantly enriched. The mean value and standard deviation for qPCR data were calculated. The normal distribution of qPCR data was validated via Shapiro–Wilk Tests. The significance of pairwise gene comparisons was conducted with parametric t-tests or non-parametric Mann–Whitney Tests (both two-tailed), depending on the data distribution.

## Results

### Purity of pancreatic stellate cells

**Figure 2:**
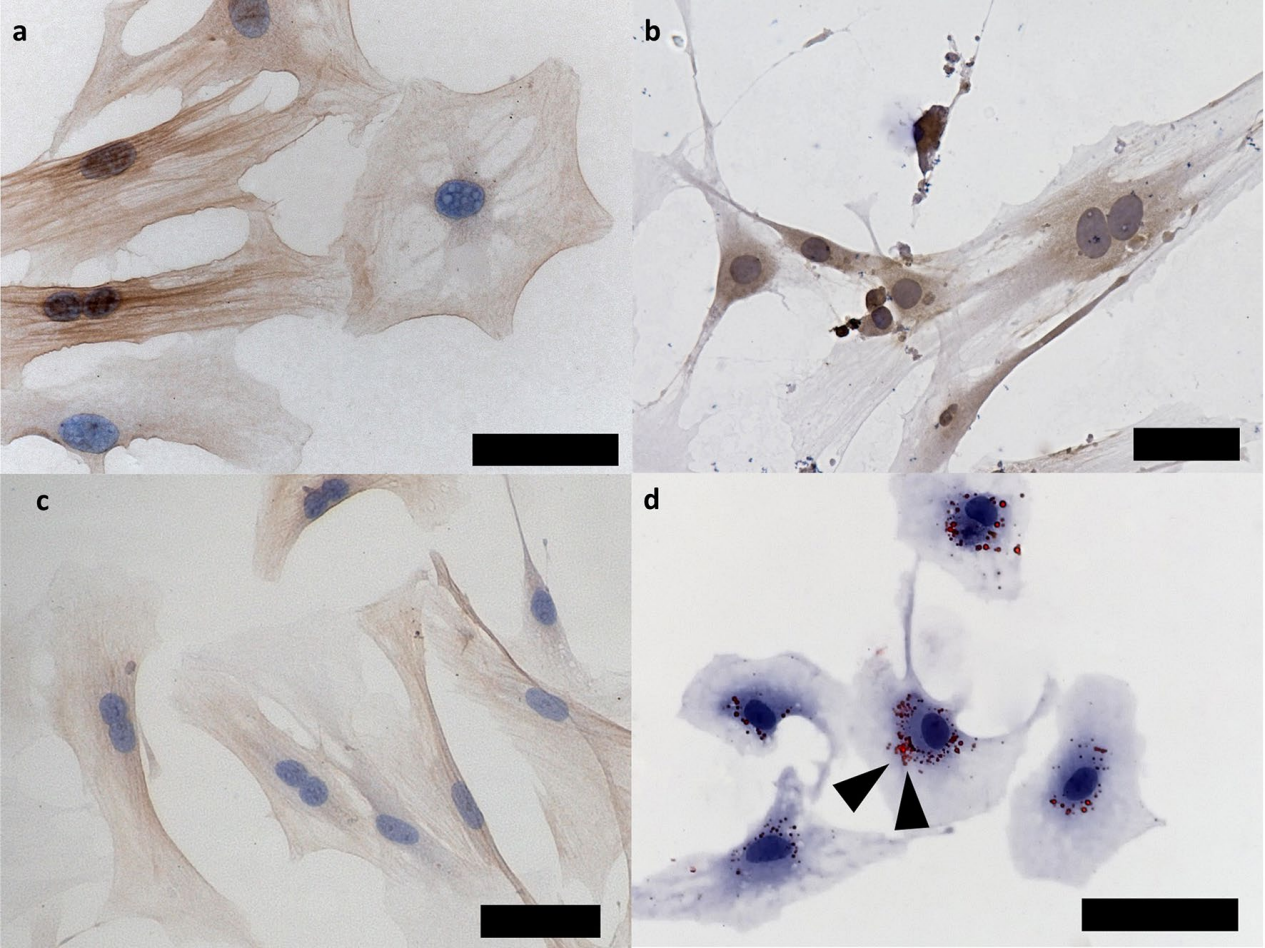
Immunocytochemistry of murine PSC. PSC show constant positive staining for α-SMA (**a**), GFAP (**b**) and Vimentin (**c**). Oil red O staining shows red cytoplasmic lipid droplets (black arrows, **d**). magnification 40x, scale bars 50 µm.

PSC were isolated from C57BL/6 mice pancreata by Nycodenz gradient centrifugation^13^, and purity was verified by morphologic features and immunostaining. The initial round shape, shortly after isolation, changed over time to a star-like shape with irregularly formed cell extensions. PSC were positive for αSMA, GFAP, and Vimentin (Fig. 2a–c). Oil red O staining revealed the characteristic cytoplasmic lipid droplets in PSC^4^ (Fig. 2d). Absence of the epithelial marker CK19^21^ confirmed that there was no contamination of PSC cultures (Fig. S2).

### *Electron* microscopy

**Figure 3:**
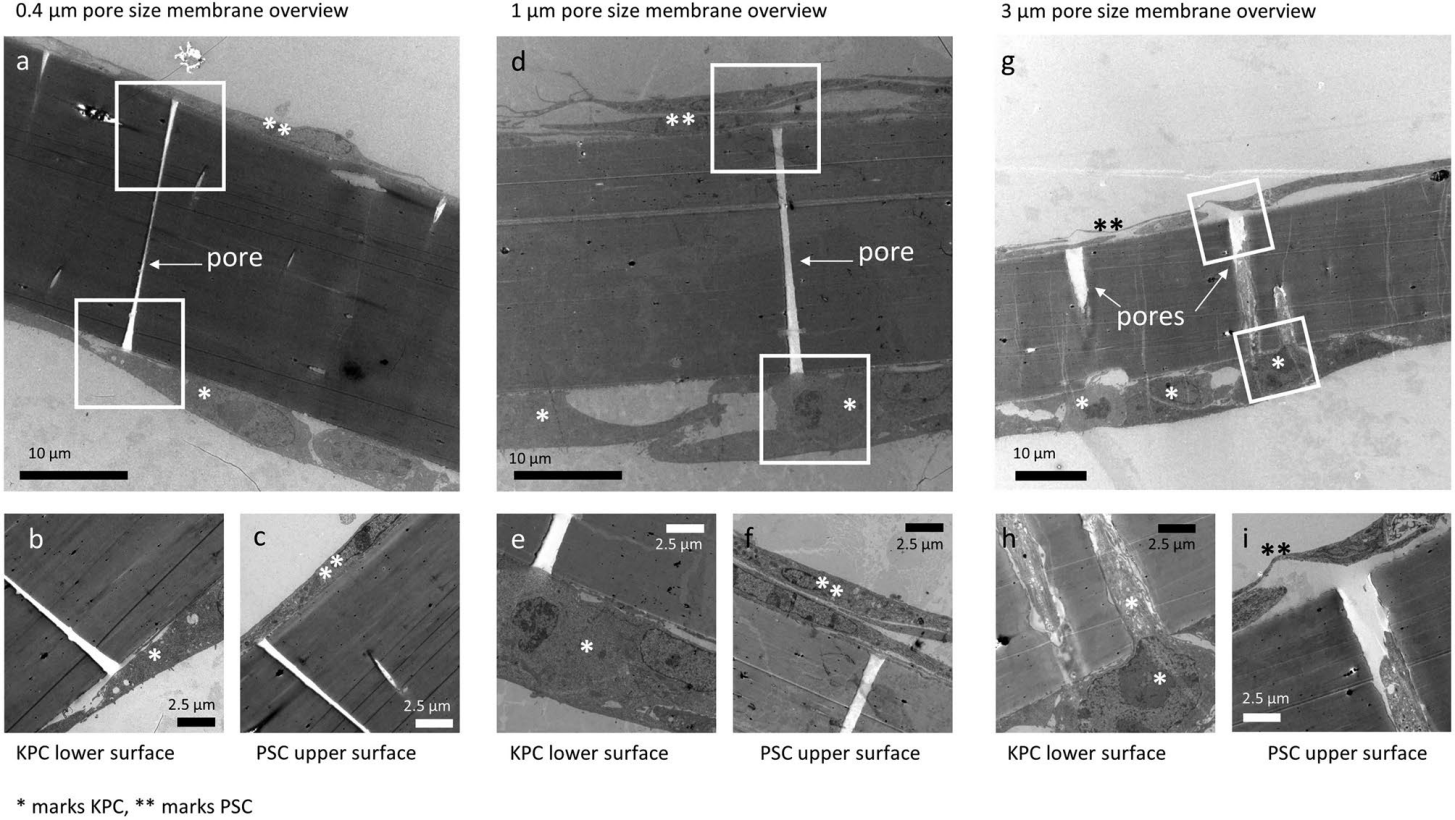
Electron microscopy images of the cell culture-insert membranes in different sizes after 72 h co-culture. Overview of membrane with 0.4 µm pores (**a**) and a magnification of the same picture showing the membrane lower surface with KPC cells (**b**) and the upper surface with PSC (**c**). Overview of membrane with 1 µm pores (**d**) and a magnification of the same picture showing the membrane lower surface with KPC cells (**e**) and the upper surface with PSC (**f**). Overview of membrane with 3 µm pores (**g**) and a magnification of the same picture showing the membrane lower surface with KPC cells, which are reaching inside the pore (**h**), and the upper surface with PSC (**i**).

Electron microscopy was performed to rule out the possibility that PSC and cancer cells stay in physical contact through the membrane's pores. PSC show a typical flat elongated growth pattern, whereas KPC cells are apparent as cubic, irregular shaped, dense structures on the membrane (Fig. 3 a, d, g). KPC cells present a prominent, large nucleus with a higher nuclear–cytoplasmic ratio than PSC. Furthermore, PSC can be distinguished from KPC by mitochondria with a dark matrix. Co-cultures and electron microscopy imaging were carried out for three different pore sizes: 0.4 µm, 1 µm, and 3 µm. As expected in co-cultures with 0.4 µm pores, no cells were able to extend into the pores (Fig. 3a–c). In 3 µm pore co-cultures (Fig. 3g–i), PSC were found on the bottom of the membrane and, vice versa, cancer cells on top of the membrane, thus indicating a movement of cells along the membrane's pores in the different compartments (Fig. S3). Lastly, in co-cultures with 1 µm pores, cells started to extend in some pores but did not reach in any observed section the middle line. Altogether, no physical contact of PSC and KPC cells in co-cultures with 1 µm pore size was detected (Fig. 3d–f). Hence, the pore size of 1 µm was chosen for the following experiments.

### RNA sequencing data quality, mapping results, and correlation of biological replicates

RNA sequencing at 24 h and 72 h was performed from PSC co-cultured in standard co-culture with KPC cells and from PSC co-cultured in inverse co-culture with KPC cells. The base error rate was in all samples < 0.03%. After filtering raw reads, > 89.80% were clean reads. The GC content distribution ranged between 52.72% and 50.90% in all sequenced samples. The quantity of total mapped reads or fragments (TMR) was in all samples > 90%. The percentage of multiple mapped reads (MMR) was in all samples < 10%. The distribution of reads in the reference genome was mainly exonic (> 82.85%) and only in small parts, intronic or intergenic. Correlation between biological replicates was calculated using the Pearson correlation coefficient corresponding to the FPKM of each sample. The Pearson correlation coefficient of all biological replicates ranged between 0.905 and 0.990 (M = 0.964, σ = 0.018).

### Inverse co-culture and standard co-culture cause different transcriptome changes in PSC

**Figure 4:**
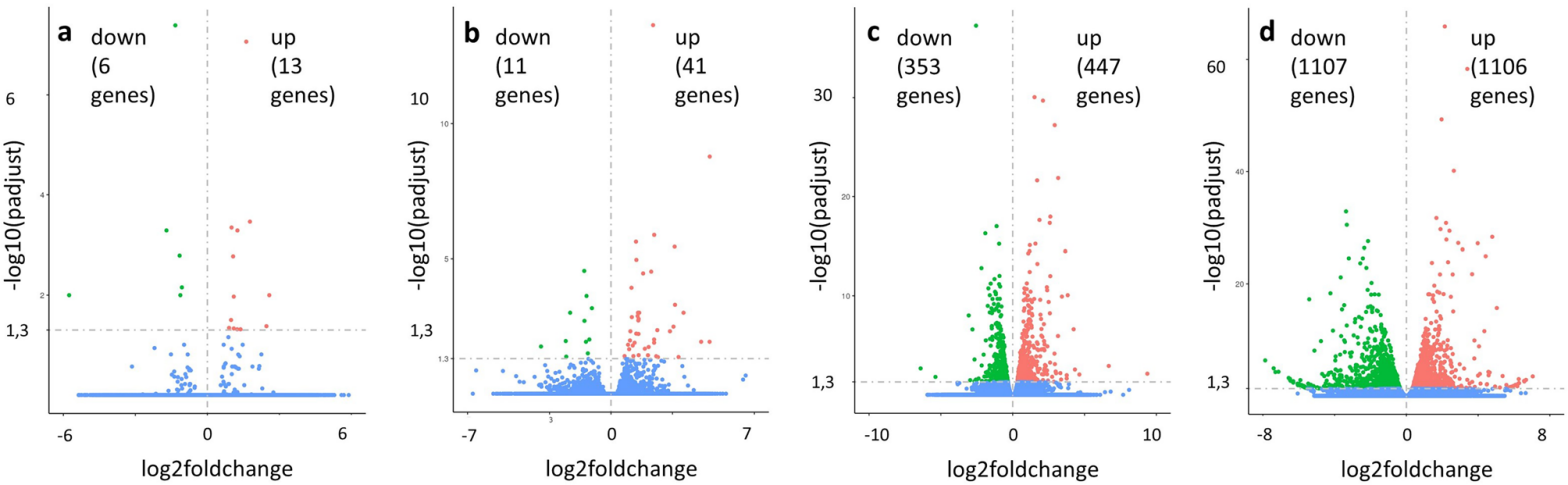
Volcano plots of up- and down-regulated DEG of PSC compared to control-PSC at 24 h and 72 h. DEG of PSC in standard co-culture at 24 h (**a**), DEG of standard co-culture at 72 h (**b**), DEG of inverse co-culture at 24 h (**c**), and DEG of inverse co-culture at 72 h (**d**).

**Figure 5:**
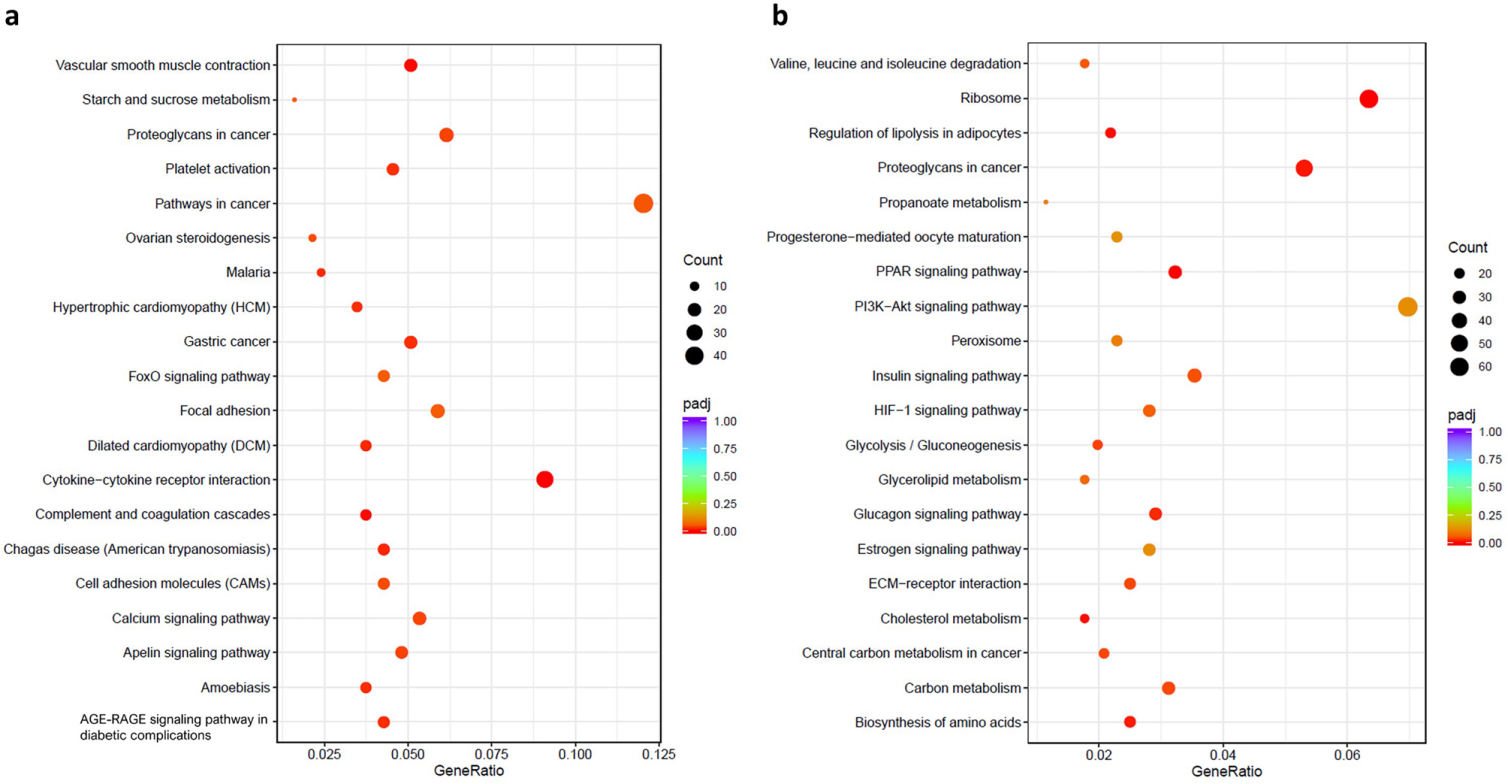
Visualisation of enriched KEGG-pathways (analysis with all DEG) in PSC of inverse co-culture. KEGG-pathways of inverse co-culture at 24 h (**a**) and 72 h (**b**).

Volcano plots display the number, significance, and logarithmic fold change of DEG of PSC of inverse and standard co-culture at 24 h and 72 h (Fig. 4a–d). In standard co-culture, 19 genes were differentially expressed at 24 h and 52 at 72 h (Fig. 4a, b) compared to mono-cultured PSC. In inverse co-culture, PSC differed stronger from their controls than in standard co-culture, resulting in a much higher number of significant DEG with 800 DEG at 24 h and 2213 DEG at 72 h (Fig. 4c, d). The number of DEG increased over time in all co-culture models, with approximately double the number of DEG at 72 h compared to 24 h. Since a high number of DEG were found in inverse co-culture, the enrichment of Kyoto Encyclopedia of Genes and Genomes (KEGG)-pathways as a functional analysis was investigated. In standard co-culture, no KEGG-pathways were significantly enriched. In inverse co-culture at 24 h, 13 (analysis with all DEG), 9 (analysis with up-regulated DEG), and 12 (analysis with down-regulated DEG) pathways were significantly enriched. At 72 h, 17 (analysis with all DEG), 4 (analysis with up-regulated DEG), and 12 (analysis with down-regulated DEG) pathways were significantly enriched. Enriched KEGG-pathways, which included up- and down-regulated DEG, are visualised in Fig. 5. At 24 h, pathways like *Cytokine-cytokine receptor interaction* and *Cell adhesion molecules* were enriched in up-regulated genes. In contrast, cytoskeleton-associated pathways (*Regulation of actin cytoskeleton*, *Focal adhesion*) were enriched in down-regulated DEG (Fig. 5a). At 72 h, cell growth-associated pathways (*Cell cycle*, *Biosynthesis of amino acids* and *Ribosome*) and cancer-associated pathways (*PI3K-Akt signalling pathway*, *MicroRNAs in cancer*, *Central carbon metabolism in cancer*) were enriched in up-regulated DEG. In contrast, Lipid-metabolism associated pathways (*Cholesterol metabolism*, *Glycerolipid metabolism*, *Fatty acid metabolism*) were enriched in down-regulated DEG (Fig. 5b). Among all enriched KEGG-pathways *Proteoglycans in cancer* was identified as a mutually enriched pathway.

**Figure 6:**
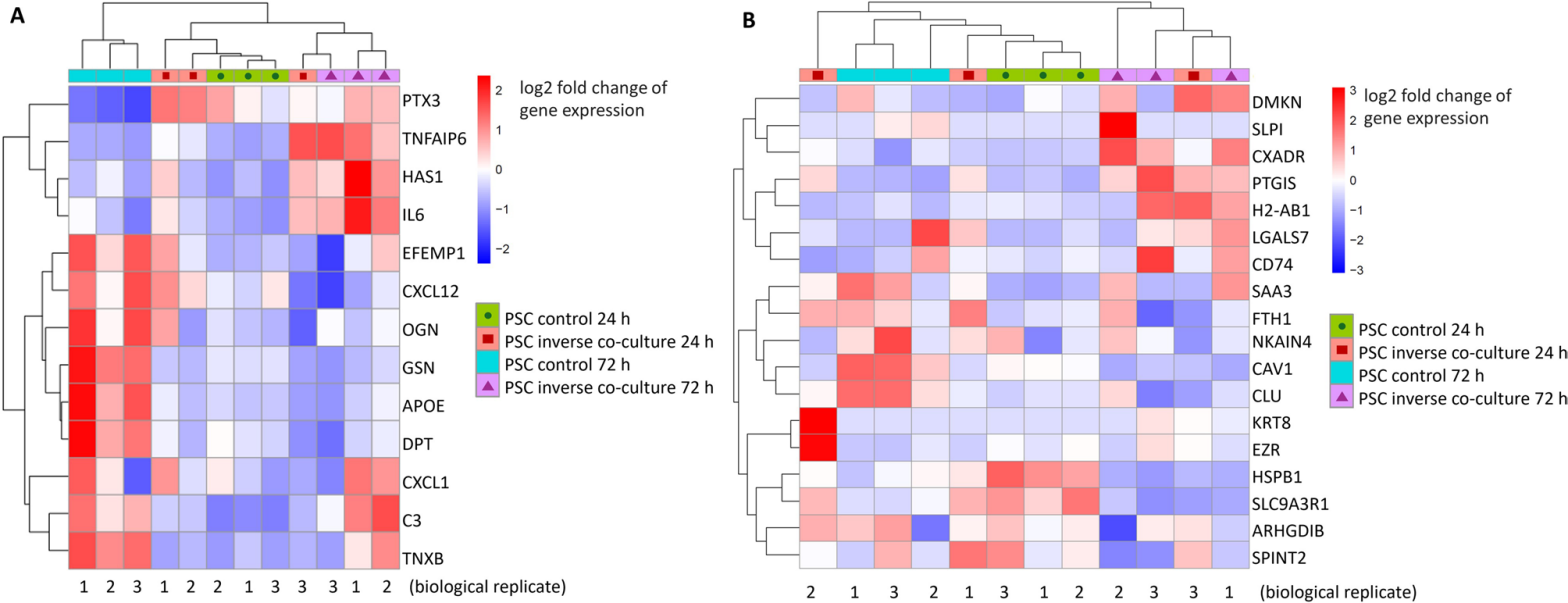
Heat maps of expression of inflammatory cancer-associated fibroblasts (iCAF) (**A**) and antigen-presenting cancer-associated fibroblasts (apCAF) (**B**) markers in PSC from KPC inverse co-culture at 24 h and 72 h. Colour (blue/red) corresponds log2 fold change of gene expression (down-/up-regulation).

Furthermore, expression of CAF signature genes in PSC of both co-culture models were investigated, to discover if PSC can be assigned to a defined CAF subtype. Distinct phenotypes of CAFs have been described: inflammatory CAF (iCAFs), located further from cancer cells and expressing cytokines, myofibroblastic CAF (myCAFs), which can be found in direct proximity to cancer cells and are characterized by the expression of αSMA^10^, and antigen-presenting CAFs, which can act in a immunomodulatory manner via activation of CD4 + T cells^22^. Quantitative expression of a set of genes already described for iCAF, myCAF and apCAF^22^, that are expressed both in humans and mice, was examined. At 72 h, PSC from inverse co-culture with KPC cells displayed changes in iCAF and apCAF marker expression (Fig. 6A,B), whereas no such changes were observed for myCAF signature genes (data not shown). Differential gene expression analysis of apCAF and iCAF genes showed no homogenous up- or down-regulation, but a heterogenous expression of genes. Upregulated iCAF signature genes in co-culture PSC included, amongst others, IL6, as a prominent iCAF marker^8^, but down-regulation for example of gelsolin and other iCAF markers. In PSC from standard culture with KPC no clustering for iCAF, myCAF or apCAF markers was observed (data not shown).

**Figure 7:**
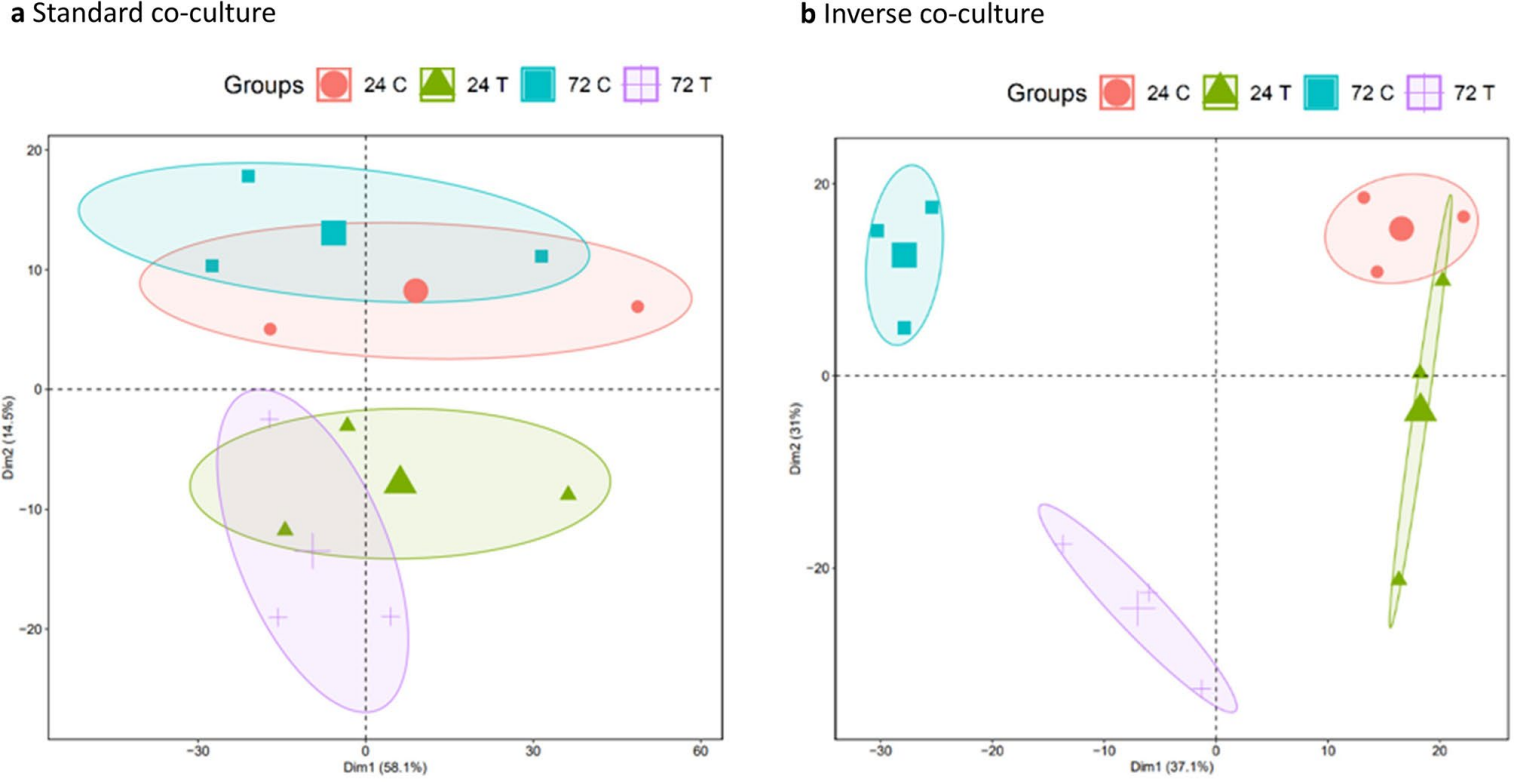
PCA of standard co-culture and inverse co-culture model. Displayed groups: control PSC at 24 h (24 C), PSC from KPC co-culture at 24 h (24 T), control PSC at 72 h (72 C), PSC from KPC co-culture at 72 h (72 T), n = 3 for each group. PCA of standard co-culture model with KPC cells (**a**) and inverse co-culture models with KPC cells (**b**).

Principal component analysis (PCA) was performed to visualize the organisation of samples of each model. In the PCA of the standard co-culture model, treatment groups are distinguishable from control groups while showing an overlap with the respective other time point (Fig. 7a). The PCA of inverse co-culture shows fewer similarities between the treatment and the control group over time. There is no overlapping between the treatment groups (Fig. 7b). Comparing PCAs of standard co-culture and inverse co-cultures, the standard co-culture model displays a lower variation over time, as an overlap of treatment groups of 24 h and 72 h is observed in standard co-culture. These observed similarities between treatment and control groups are probably due to less intense impact of KPC on PSC when cultured in two different compartments and separated further. Inverse co-culture shows a greater variation over time, therefore, especially at 72 h treatment and control groups are clearly spatially separated. Changes of gene expression caused by inverse co-culture are stronger, probably due to intensified paracrine KPC-PSC interactions when cultured in proximity.

PCA of control PSC only was conducted to confirm reproducibility of PSC controls (Fig. S4). This analysis shows that controls of 24 h and 72 h of both co-culture models group mostly based on the time point of analysis, which is expected due to identical culture-conditions.

**Figure 8:**
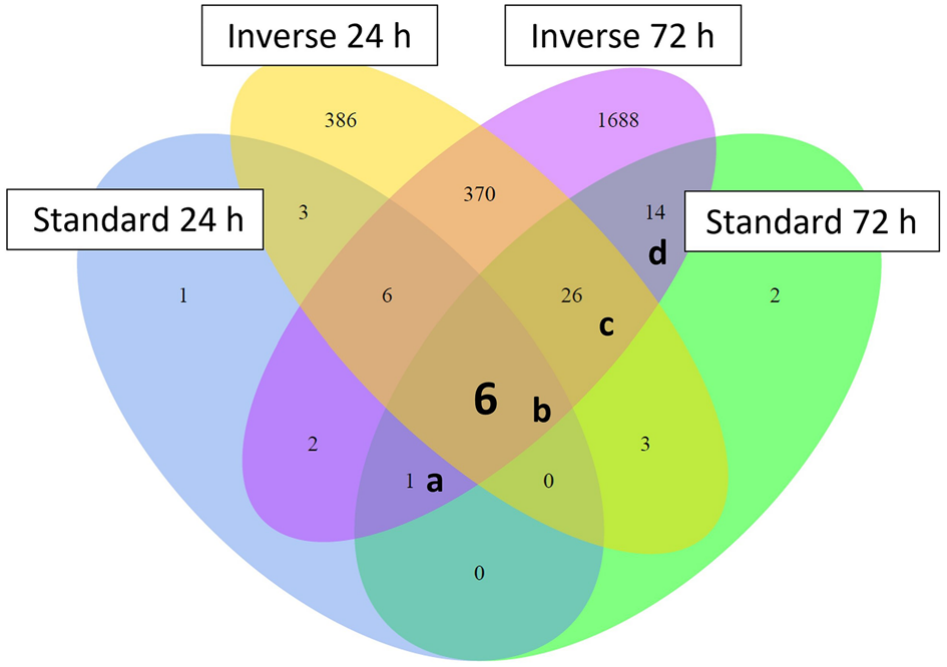
Venn diagram of DEG in PSC in inverse and standard co-culture after 24 h and 72 h co-culture with KPC cells. Genes of fields a, b, c, and d are listed in Table 2.

The results mentioned above demonstrate differences in gene expression in PSC of inverse and standard co-culture. To identify mutually expressed genes, which may play a key role since they are expressed or activated under different circumstances, DEG of all time points of standard and inverse co-culture were compared via a Venn diagram (Fig. 8). Overall, 6 genes were expressed in common at both time points, in both co-cultures, independent of the experimental set up. These 6 genes are Versican (VCAN), SH3 And Cysteine Rich Domain 2 (STAC2), Cystin 1 (CYS1), Carbohydrate Sulfotransferase 11 (CHST11), 6030408B16Rik and Acyl-CoA Synthetase Long Chain Family Member 4 (ACSL4) (Fig. 8b, Table 2). In inverse co-culture at both time points and standard co-culture at 72 h 26 genes were mutually expressed, among them genes of the KEGG pathway *Proteoglycans in cancer*, like Fibronectin 1 (FN1), Hepatocyte Growth Factor (HGF), Epiregulin (EREG) and Tissue Inhibitor of Metalloproteinases 1 (TIMP1) (Fig. 8c, Table 2). 14 genes were exclusively differentially expressed in inverse and standard co-culture at 72 h (Fig. 8d, Table 2).

**Table 2:** DEG in PSC in inverse co-culture and standard co-culture as represented by the Venn diagram (Fig. 8).

Field/intersection	Gene name(s)
**a** (standard 24 h and 72 h and inverse 72 h)	TNFSF15
**b** (standard and inverse 24 h and 72 h)	VCAN, STAC2, CYS1, CHST11, 6030408B16Rik, ACSL4
**c** (inverse 24 h and 72 h and standard 72 h)	EREG, ELN, TIMP1, CDR2L, LFNG, FN1, BMP4, HGF, FRZB, MCFD2, SMIM3, JAG1, UGCG, CCDC85A, MGARP, PQLC1, RASA4, SDC4, UGDH, SOCS5, INSC, SLC2A3, PI4K2B, PTPRJ, F5, PCDH10
**d** (invers and standard 72 h)	CHRDL2, GPR35, GAS6, NDUFA4L2, SPON2, PTX3, RTN1, 4930447F24Rik, IVL, CREB3L3, MARCH3, A730049H05Rik, GM13431, WSCD2

### PPI-network analysis

**Figure 9:**
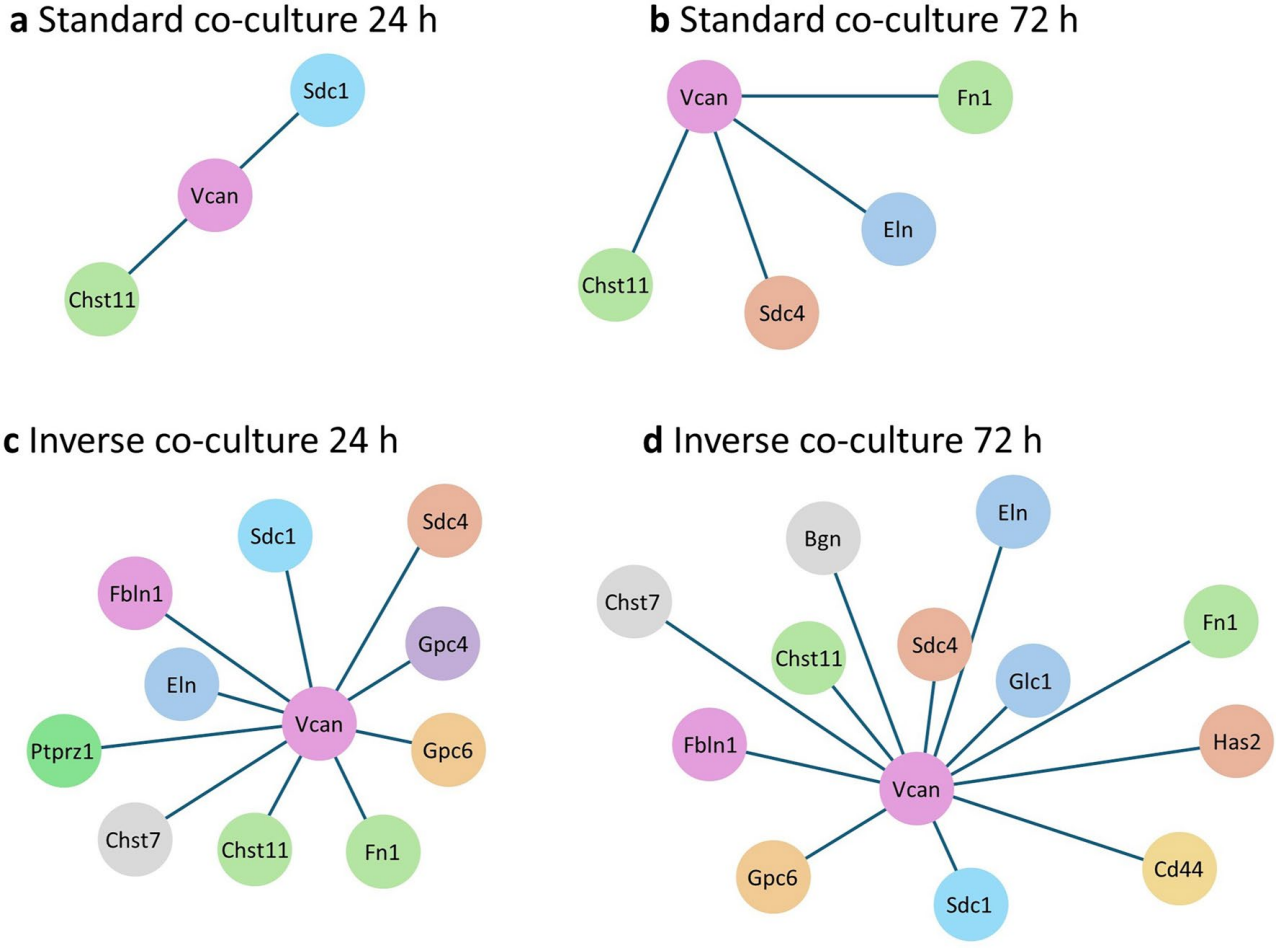
String PPI-networks of standard and inverse co-culture with VCAN first neighbor genes. First neighbor genes of VCAN in Standard co-culture at 24 h (**a**) and 72 h (**b**) and in inverse co-culture at 24 h (**c**) and 72 h (**d**).

Protein–protein interaction (PPI)-network analysis of both models and time points was performed via STRING service^23^ and Cytoscape software^17^. PPI-networks showed an increased complexity over time and with a reduced distance of cancer cells and PSC. Inverse co-culture at 72 h had the most genes enriched in the PPI-network. Due to its upregulation in all co-culture models at both time points, VCAN PPI-networks were analysed. Only VCAN first neighbor genes were selected to narrow down probable relevant genes, and the confidence cutoff was set at 0,8 (Fig. 9a–d, Table 3). VCAN PPI-networks of both co-cultures shared multiple genes such as CHST11, FN1, and syndecan 4 (SDC4). CHST11 and VCAN are up-regulated in both co-cultures at both time points (Table 4).

**Table 3:** Subscores of first neighbor genes of VCAN in string PPI-network of all co-culture models (Fig. 9).

Node 1	Node 2		String Co-expression	Experimentally determined interaction	String database score	String textmining
VCAN	FN1	Fibronectin 1	0.132	0	0.949	0.944
	CHST1	Carbohydrate sulfotransferase 11	0.079	0	0.946	0.469
	SDC1	Syndecan 1	0	0	0.952	0.446
	ELN	Elastin	0.062	0	0.891	0.888
	SDC4	Syndecan 4	0	0	0.949	0.515
	CD44	CD44 antigen	0.069	0.164	0.988	0.164
	PTPRZ1	Protein tyrosine phosphatase receptor type Z1	0.061	0.047	0.801	0.795
	CHST7	Carbohydrate sulfotransferase 7	0	0	0.9	0.239
	FBLN1	Fibulin 1	0.088	0.089	0.97	0.967
	GPC6	Glypican 6	0.062	0	0.9	0.314
	GPC4	Glypican 4	0.077	0	0.9	0.302
	BGN	Biglycan	0.087	0.067	0.909	0.851
	GPC1	Glypican 1	0.073	0	0.941	0.425
	HAS2	Hyaluronan Synthase 2	0.12	0	0.821	0.805

**Table 4:** RNASeq differential gene expression in PSC of selected VCAN PPI-network genes in different co-culture models, n. s.: not significantly differentially expressed.

		Inverse 24 h (log 2 foldchange)	Inverse 72 h (log 2 foldchange)	Standard 24 h (log 2 foldchange)	Standard 72 h (log 2 foldchange)
VCAN	Versican	1.25	1.26	1.60	1.60
CHST11	Carbohydrate Sulfotransferase 11	1.06	1.27	1.07	1.22
SDC4	Syndecan 4	0.70	1.55	n. s	1.10
FN1	Fibronectin 1	1.10	1.60	n. s	1.30

### Quantitative RT-PCR

**Figure 10:**
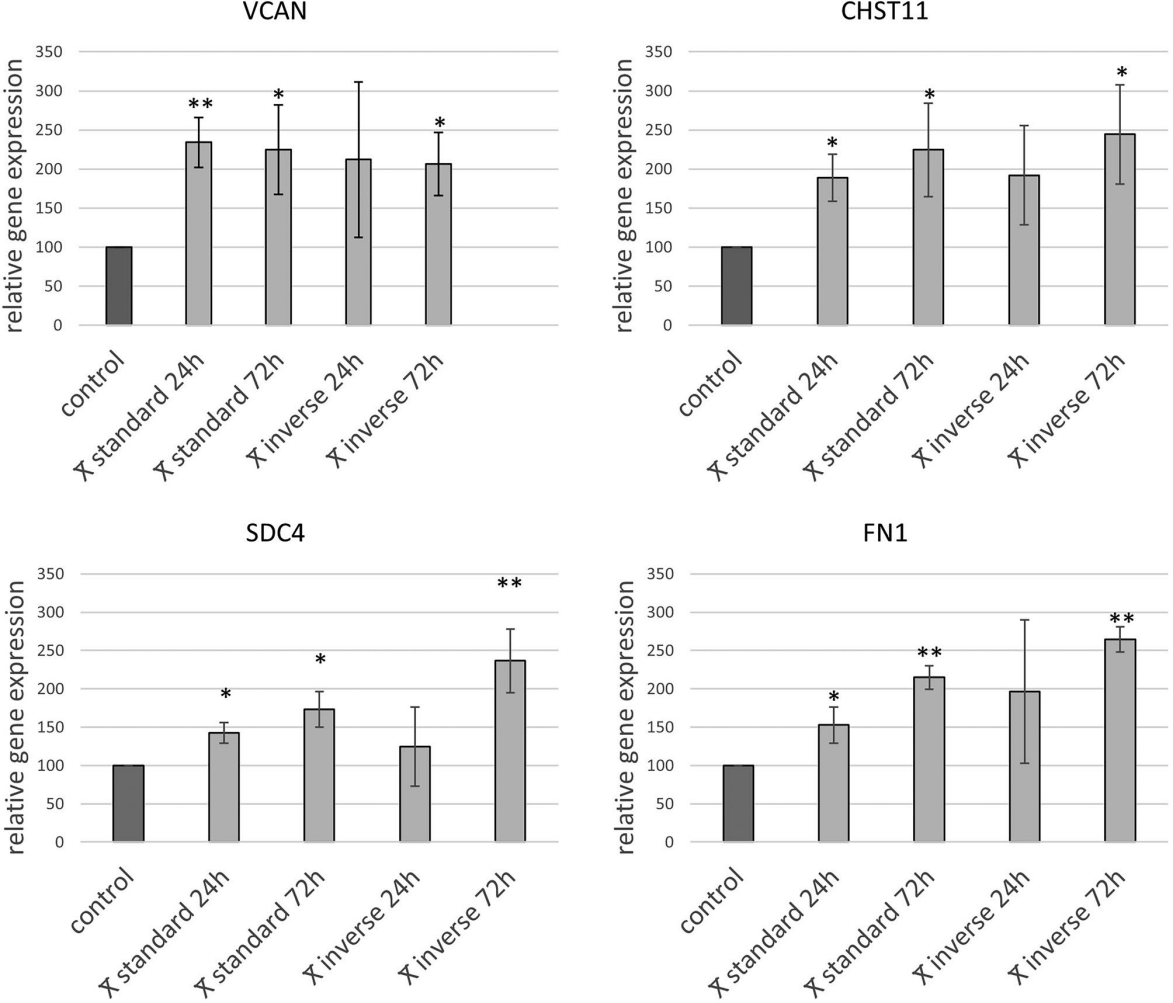
Comparative analysis of qPCR abundances of VCAN, CHST11, SDC4, and FN1 expression in PSC in standard and inverse co-culture at 24 h and 72 h; mean (n = 3) + /- SD; *p < 0.01; **p < 0.001.

To validate the results of the above-described analysis, qPCR of selected genes was performed. Corresponding to the differential gene analysis of RNASeq data, the genes VCAN, CHST11, SDC4, and FN1 were significantly up-regulated compared to controls in most time points but at 24 h in Inverse co-culture (Fig. 10). The gene expression of the respective genes in control PSC over time has an influence on interpreting and understanding the data, as the values of the gene expression of the treated PSC are normalized by the values of the corresponding control PSC. Therefore, a comparative quantification analysis of the controls of each co-culture triplicate comparing 24 h controls against 72 h controls was performed (Fig. S5). There were no significant differences in gene expression in between 24 h control PSC and 72 h control PSC for the respective genes (VCAN, SDC4, FN1, CHST11).

### Immunocytochemistry

**Figure 11:**
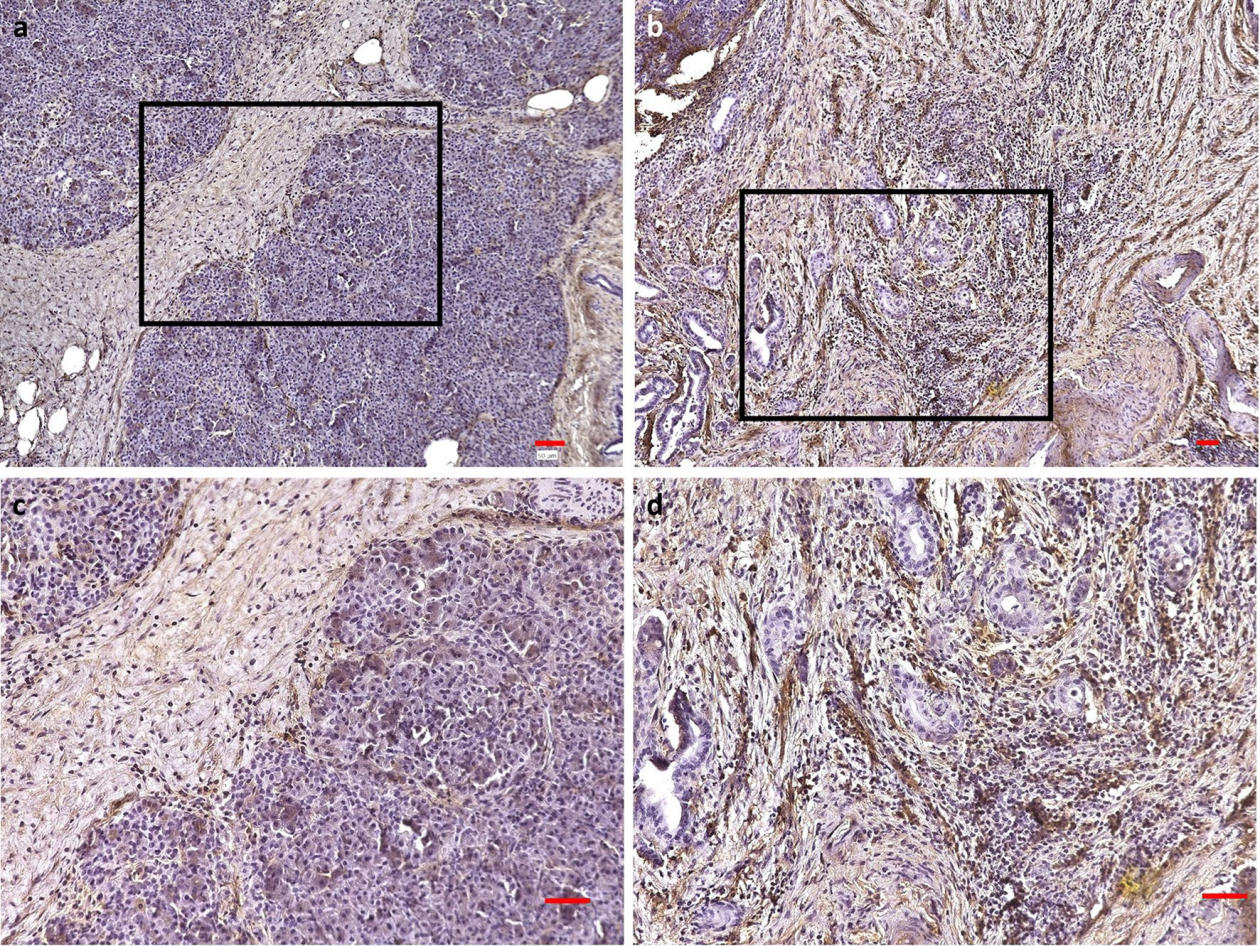
VCAN immunocytochemistry. VCAN immunocytochemistry in human normal pancreas next to cancer (**a**, **c**); **c** is magnified inset of **a** (magnification 20x). VCAN immunocytochemistry of human pancreatic cancer tissues (**b**, **d**); **d** is magnified inset of **b** (magnification 20x); scale bars 50 µm.

VCAN immunocytochemistry was performed on human PDA samples (n = 3) and normal pancreas (n = 3) next to cancer tissues. All PDA samples showed an enhanced stromal VCAN accumulation compared to normal pancreas, with a lower stromal expression (Fig. 11a–d).

## Discussion

Given the increasing incidence, very low five-year survival rates^1^, and often late-stage diagnosis^2^, it is crucial to gain a more comprehensive understanding of the initiation and progression of PDA. PSC not only play a role in maintaining a healthy pancreatic stroma^4^ but also serve as a source of CAF within the PDA stroma^24^. Therefore, this work aimed to examine interactions between PSCs and cancer cells under various conditions to better understand their paracrine crosstalk. It has been shown previously in murine models that CAF adopt different phenotypes influenced by whether they have direct contact with cancer cells or only via paracrine effects^8^. In this work, the proximity of both cell types during paracrine communication was highlighted as a new parameter of this complex relation. Therefore, two different paracrine co-culture models with PSC and KPC cells, standard co-culture and inverse co-culture, differing by the distance of cells, were established.

PSC cannot be assigned to previously described CAF subgroups. Corresponding with findings in mice that juxtacrine interaction is needed for the induction of a myCAF phenotype^8^, PSC of both co-cultures did not show a myCAF gene signature. In inverse co-culture, enhanced expression of a subset of iCAF/apCAF markers and clustering for the respective gene sets in heat maps was observed at 72 h, even though there was also a subset of iCAF/apCAF markers with decreased expression. Co-culture periods were relatively short, as time points of RNA sequencing were chosen to investigate early interactions of PSC and cancer cells. Therefore, the period of interaction and communication might not have been long enough to induce a distinct CAF phenotype. This hypothesis is supported by the clustering of samples at 72 h, which is not yet evident at 24 h. Another reason for the heterogenous up-and down-regulation of genes in the respective gene sets might be a mixed CAF type, expressing some iCAF and some apCAF markers or a mix of iCAF and apCAF cells.

As a second approach to quantify the abundance of RNA transcripts, i.e. qPCR was conducted for selected genes. Results from RNA sequencing data were concordant with qPCR results, as significant up-regulation for investigated genes was observed at most the points, except for 24 h in inverse co-culture. Additionally, as no difference in expression of the analyzed genes between both controls, 24 h and 72 h, was found, this confirms that the observed trend in the comparison of treated PSC is caused by the treatment itself. Whereas direct comparison of values of RNA sequencing data and qPCR values is not constructive, qPCR can be a valuable tool to confirm observed trends in RNA Seq data, especially if differences of expression of selected genes are small^25^. Altogether findings of qPCR provide an independent validation of a part of RNA sequencing results.

RNA sequencing of PSC after 24 h and 72 h co-culture revealed that changes in PSC gene expression profiles occur after 24 h and increase over time in both co-cultures. The maximum time of co-existence of PSC and KPC cells differed in standard co-culture and inverse co-culture due to technical reasons; therefore this has to be taken into consideration interpreting the results. It is shown, however, that already after 24 h of inverse co-culture the differential gene expression in PSC was many times over the differential gene expression of PSC cultured for 72 h in standard co-culture, suggesting that the specific co-culture method is the dominant driver of gene expression. A reduced distance between PSC and KPC cells led to a 40 times higher number of differentially expressed genes in PSC. Among relevant enriched signalling pathways such as cytokine interaction, cell growth, and proteoglycan metabolism in inverse co-culture, the pathway *Proteoglycans in cancer* was mutually enriched at 24 h and 72 h. Differential gene expression analysis revealed six genes that were expressed in common in PSC in both co-cultures at both time points. As genes expressed in common, independent of the experimental setup, might be very robust in activation and essential for early processes in the tumor microenvironment, these six genes were further investigated. The proteoglycan VCAN and the enzyme CHST11 were therefore identified as potential key genes.

VCAN is a chondroitin sulfate proteoglycan and a component of the extracellular matrix involved in various physiological and pathological biological processes. Expression and modification of VCAN are regulated by different pathways such as Wnt/ß-catenin and multiple factors such as interferons, growth factors, and miRNAs^26^. CHST11 is an enzyme of the CS proteoglycan biosynthesis, which can be found in the Golgi apparatus and facilitates the sulfation of chondroitin at the C4 position of N-acetylgalactosamine (C4-GalNAc), leading to the formation of the CS subtype CS-A, one of six different CS subtypes^27^. The sulfation pattern of VCAN in PDA differs from normal pancreas tissue. In PDA, 6-sulfated disaccharides are predominant, whereas in a normal pancreas, 4-sulfated disaccharides build most of the sulfation pattern^28^. In this study, CHST11, which catalyzes the creation of 4-sulfated disaccharides, was up-regulated. CHST1, which catalyses the creation of 6-sulfated disaccharides^28^, was not significantly altered. In previous studies chemical analysis of human PDA tissue revealed an up to 27-fold increase of VCAN with an altered sulfation pattern, shifting towards a richer chondroitin sulfate (CS) content of side chains. These changes could create a tumor-permissive environment, as CS chains are less likely to decelerate tumor cell migration than glycosaminoglycans like dermatan sulfate^28^. The abundance of CS chains in PDA compared to normal pancreas could also explain the elevated levels of CHST11, as the overall content of 4-sulfated disaccharides was much higher in PDA than in normal pancreatic tissue due to a higher CS content. Furthermore, the temporal dynamic of changes in the VCAN PDA sulfation pattern has not been investigated in detail yet and could play a role in the elevated CHST11 expression observed in this work. Interestingly, VCAN expression was consistently up-regulated throughout the observed time, at 24 h and 72 h, in RNA seq as well as in qPCR data, not showing an in- or decreased tendency of expression. This finding shows that VCAN upregulation begins as early as 24 h after co-culture with cancer cells. Whether a stronger upregulation of VACN occurs after extended co-culture time needs to be investigated in the future.

Carbohydrate sulfotransferases, such as CHST7, CHST11-13, or CHST15, have been suggested to be relevant in developing and progressing multiple types of cancer, as in PDA. In contrast, the exact roles of the enzymes in the PDA context have yet to be determined^29^. CHST11 may be important in tumor-associated VCAN sulfation patterns, especially in early stromal changes. In agreement with this hypothesis, CHST11 was recently found to be involved in a CAF signature of 12 genes that correlates with the overall survival of PDA patients and predicts a poor response to chemotherapy^30^. Further investigation is required to explore how and whether the upregulation of CHST11 is attributed to specific post-translational tumorigenic modifications of VCAN.

Fn1, as part of the VCAN PPI-network and up-regulated at 24 h in inverse co-culture, has been identified in a bioinformatic analysis of human PDA pathogenesis as one of the potential hub genes^31^. Fn1 is a glycoprotein of the ECM which plays a role in cell migration and adhesion processes^32^. In human PDA, Fn1 is the primary element of the tumour matrix^33^.

SDC4, as another up-regulated member of the VCAN-PPI network, has already been described in VCAN-PPI networks of breast cancer^34^. SDC4 is a transmembrane heparan sulfate proteoglycan that plays a role in extracellular matrix processes such as cell adhesion, organization of cell cytoskeleton, and cell motility. While Sdc4 is not present in PDA cells, upregulation in activated PSC was described. Syndecans also can influence tissue stiffness^35^, a notable concern in PDA, given the characteristic dense stroma associated with the disease.

The elevated levels of VCAN in this work are consistent with work from Emmerich et al., where even in the earliest stages of PDA in mice, VCAN production was detected by immunohistochemistry in epithelial and stromal cells^36^. Corresponding to these findings, in this work, the immunohistochemistry of human PDA samples showed an enhanced VCAN expression compared to the healthy pancreas. Both epithelial and stromal cells can be sources of VCAN. VCAN derived from myeloid cells acts anti-inflammatory, whereas VCAN from stromal cells is part of the inflammatory response that occurs in most cancers by interacting with immune cells, chemokines, and growth factors^26^. This study does not provide evidence of whether the increased VCAN gene expression in PSC has a protective or cancer-permissive effect; hence, obtaining a better understanding of the pro- and anti-tumorigenic functions of VCAN is essential. Despite the abundance of identified potential key genes in PDA bioinformatic studies, VCAN was consistently described as one of the relevant hub genes in PDA^31,37^, which emphasizes the possible key role of VCAN.

As VCAN is part of various structures in the human body, it would be interesting if specific PDA-modified VCAN variants exist, if there is shedding into the bloodstream, and how it could be detected and even be used as a biomarker. For a more detailed understanding of PSC-KPC crosstalk, it would have been beneficial to conduct RNA sequencing on PSC and KPC cells. The analysis of VCAN expression and VCAN PPI networks in KPC cells would have helped to gain a deeper insight into the complex interactions of cancer and stromal cells.

In conclusion, this study shows the importance and influence of the distance of cells to each other regarding changes in gene expression profiles. VCAN was identified as one of the potential key genes altered through early PSC cancer cell crosstalk, and further studies investigating the role of VCAN in PDA initiation and progression are needed.

### Supplementary Information

Supplementary Figures.

## Data Availability

All data generated or analyzed during this study will be provided by the corresponding author (A.Z., anais.zourelidis@uk-halle.de) at reasonable request.
