# Supplementary material for: Distance-depending transcriptome changes of pancreatic stellate cells in paracrine pancreatic ductal adenocarcinoma co-culture models
Source: Sci Rep. 2024 Aug 4;14:18030. doi: 10.1038/s41598-024-68148-6 (PMC11298529; doi:10.1038/s41598-024-68148-6)
Supplement: Supplementary file 1 — Supplementary Figures. [file 41598_2024_68148_MOESM1_ESM.pdf]

Distance-depending transcriptome changes of pancreatic stellate cells in paracrine pancreatic ductal adenocarcinoma co-culture models

Anais Zourelidis [1] *, Bogusz Trojanowicz [1], Yoshiaki Sunami [1], Gerd Hause [2], David Vieweg [1], Jörg Kleeff [1]

Affiliations

1 Department of Visceral, Vascular and Endocrine Surgery, University Hospital Halle, Martin-Luther-University Halle-Wittenberg, Halle, Germany.

2 Biocenter, Martin-Luther-University Halle-Wittenberg, Halle, Germany.

* Corresponding author: anais.zourelidis@uk-halle.de

Light microscope image of cell-culture insert membrane surface

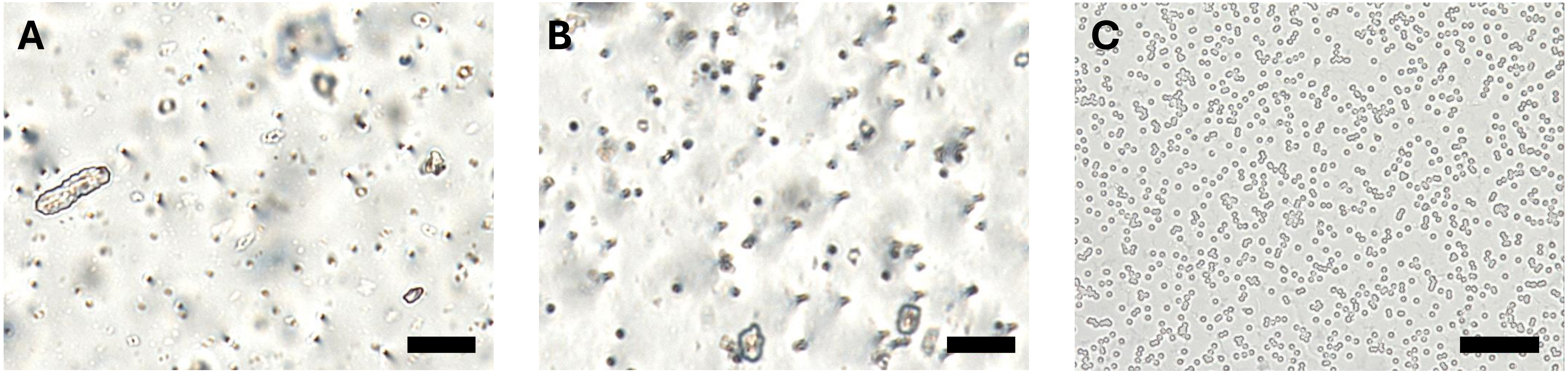

Figure S1: Light microscope image of 0.4 µm (A), 1 µm (B) and 3 µm (C) pore membranes show distribution of pores. Scale bars represent 10 µm (A, B) and 50 µm (C).

PSC immunocytochemistry

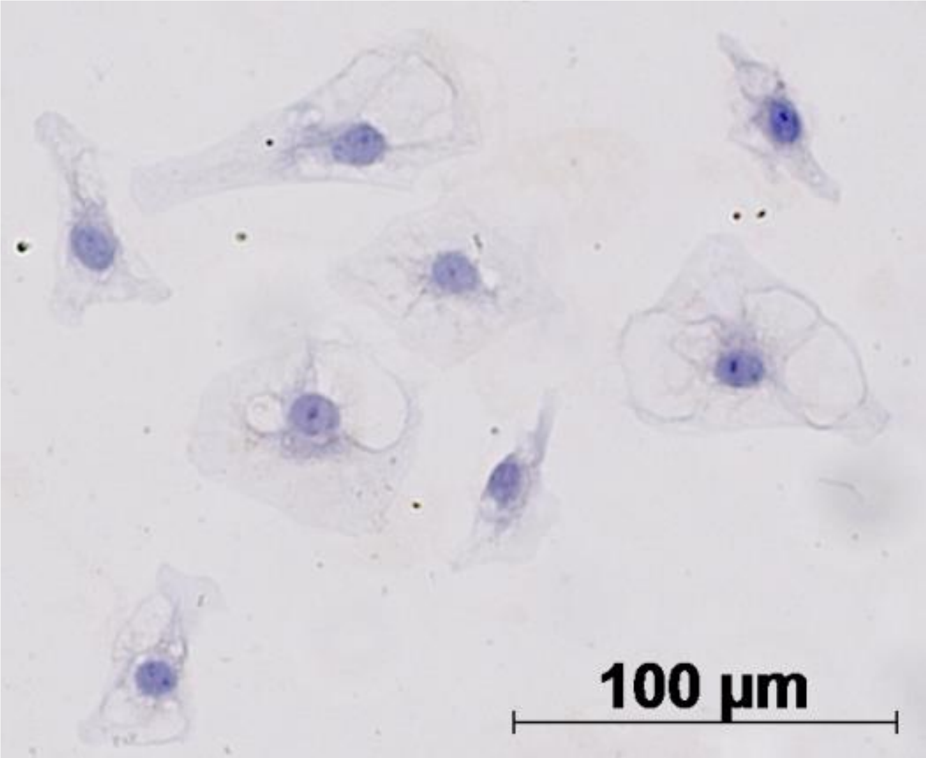

Figure S2: Immunocytochemistry of murine PSC.
PSC show no staining for the epithelial marker CK19.

PSC-KPC co-culture electron microscopy

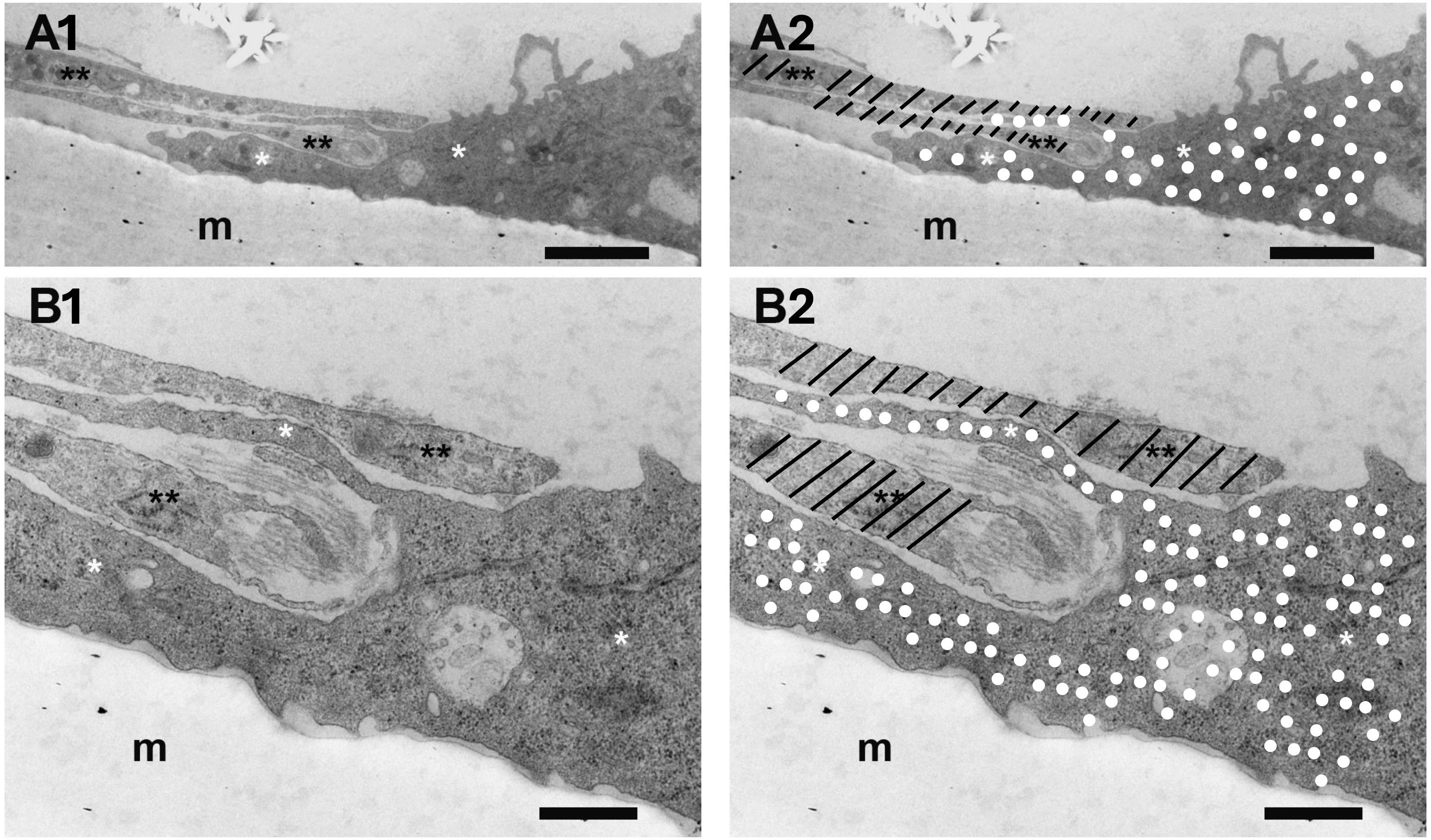

Figure S3: Electron microscopy images of inverse co-culture membrane with 3 µm pores after 72 h co-culture. An overview (A 1/2) shows that KPC (/dots) and PSC (**/stripes) are both found on top of the membrane (m). At higher magnification (B 1/2) it is visible, that protrusions of both cells are intertwined with each other. Scale bars represent 2 µm (A1/2) and 0.5 µm (B1/2).*

PCA of PSC control samples

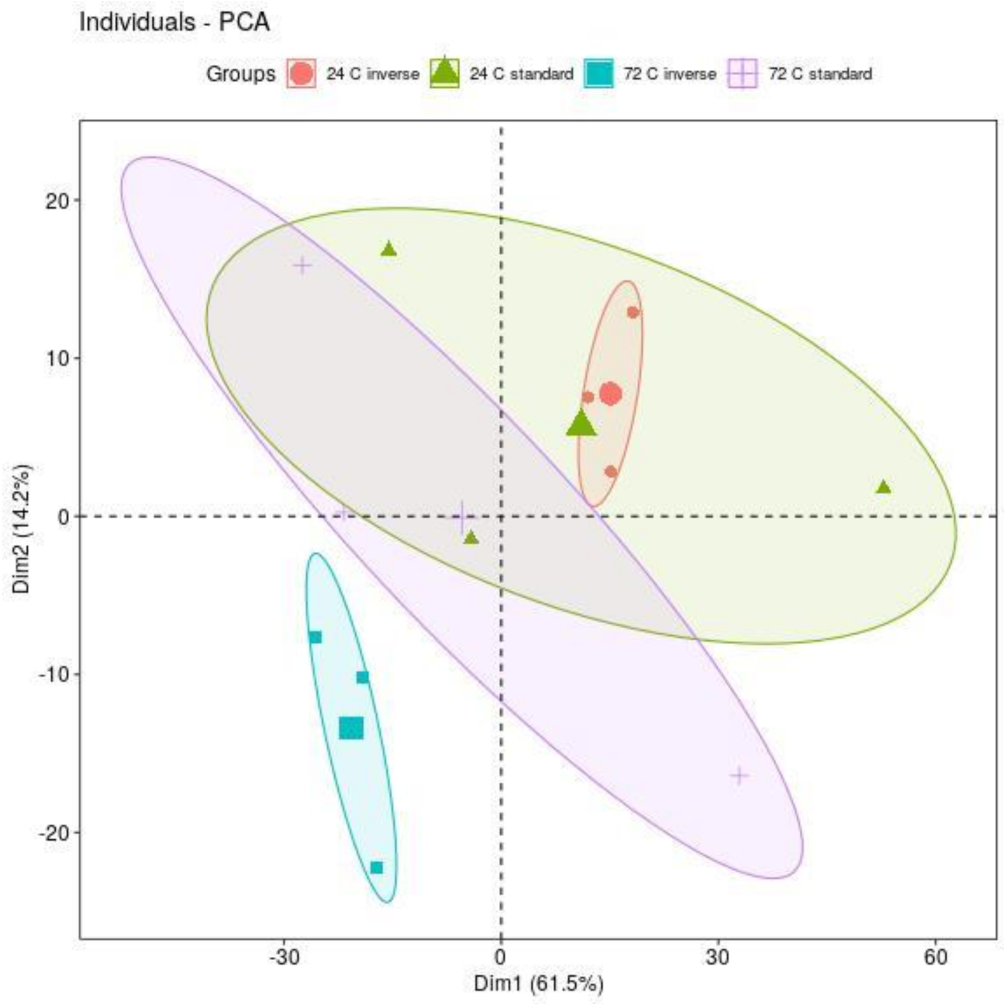

Figure S4: PCA of control PSC of standard co-culture and inverse co-culture model. Displayed groups: control PSC at 24 h of standard and inverse co-culture and control PSC at 72 h of standard and inverse co-culture (C= control), n=3 for each group.

qPCR PSC control samples

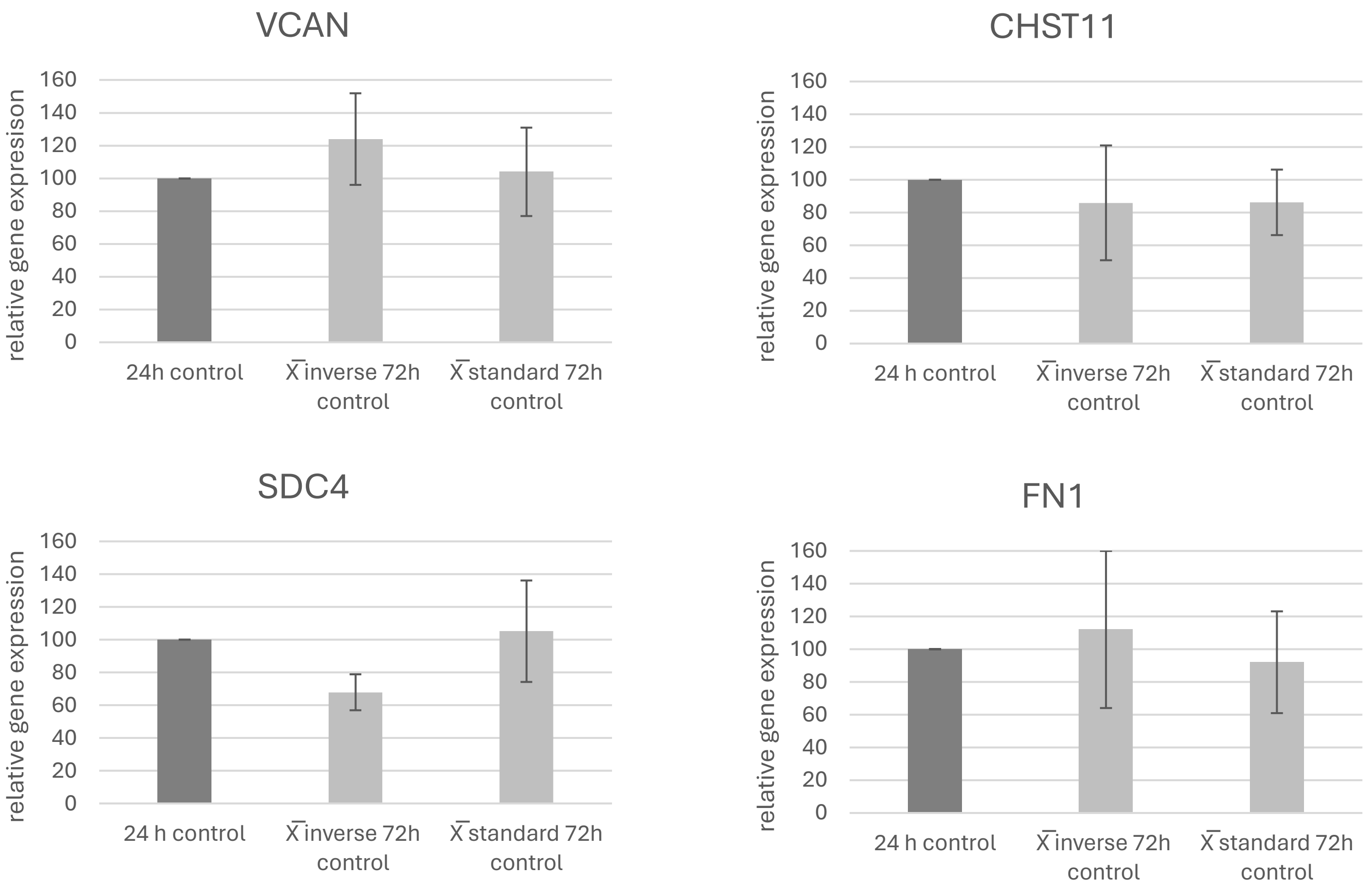

Figure S5: Comparative analysis of qPCR abundances of VCAN, CHST11, SDC4, and FN1 expression in PSC control samples in standard and inverse co-culture compared at 24 h and 72 h; mean (n=3) +/- SD; All differences are not significant (p>0.05).
